# 2017 Kermanshah Earthquake; Lessons Learned

**DOI:** 10.5249/jivr.v10i1.1049

**Published:** 2018-01

**Authors:** Alireza Ahmadi, Shahrzad Bazargan-Hejazi

**Affiliations:** ^*a*^Department of Anesthesiology, Critical Care and Pain Management, Kermanshah University of Medical Sciences, Kermanshah, Iran.; ^*b*^Charles R. Drew University of Medicine and Science (CDU) in Los Angeles, California, U.S.A.; ^*c*^David Geffen School of Medicine at UCLA, U.S.A.

On November 12, 2017 at 21:48 local time, an earthquake with a moment magnitude of 7.3 struck Ezgeleh, Kermanshah Province, Iran. It is estimated that 600 to 700 people were killed, and more than 10,000 injured. This unfortunate event gives us pause to re-examine, and thereby improve, Iran’s disaster planning, preparedness, mitigation, and program responses.

Computer-mediated technologies such as Telegram messaging service were highly beneficial. They helped mobilized local, national, and global support for victims of this disaster. Not only did social media facilitate immediate assistance for thousands of people post-earthquake, it enabled victims, friends and families to share valuable and timely information. This proved to be of immense help in rescue and relief efforts for those affected by this event. 

It is clear, however, that computers and technology alone cannot prevent or reduce injury or fatalities. In December 2003, Bam, a city in the southeast region of Iran, experienced an earthquake with a magnitude of 6.6. This destroyed nearly 90% of the city, and left approximately 40,000 dead. Post-quake assessments revealed the lack of comprehensive disaster management plans, and an inadequate emergency medical service system. Local hospitals and health facilities were found to be structurally deficient. Poor communication between and across disaster relief organizations is also blamed for the high number of casualties.^1^

It is unfortunate that what should have been learned from the Bam disaster was not actualized in the Kermanshah earthquake. Many government agencies were not well coordinated, agile, and did not have current technology and up-to-date information about the area that needed relief efforts. Perhaps as a result, earthquake victims tended to rely on those in their own family and social networks for rescue and relief, rather than on government agencies and services. This may be due in part to their distrust of, and lack of confidence in, the wisdom and efficacy of government officials in disaster response and management. The substantial damage to the newly-built Islamabad and Sar-e-Pol-e Zahab Hospitals, for example, reveals perhaps non-compliance with the “Iranian code of practice for seismic resistant design of buildings (standard no. 2800),” as well as serious deficiencies in Iranian disaster prevention and mitigation plans and measures. 

From a humanitarian perspective, we must first express our gratitude to every individual, group, organization and nation that responded so swiftly to the Kermanshah disaster, and provided generous relief and assistance. We must next consider the power and impact of technology, and what we see as infrastructure, communication and administrative shortcomings. These lead us to several important conclusions and recommendations. 

It is critical for those affected by disasters such as a major earthquake to have immediate and accurate information as to where to find shelter, and where to obtain medical and social services, food and clean water, and other essentials. A cloud-hosted, emergency information website with mapping capabilities can inform residents about the extent of damage in their area, of road closures, open petrol stations, distribution centers of essential supplies, as well as of medical and shelter sites. The recent Nepal, Haiti, and Christchurch (New Zealand) earthquakes show that combining open-source and propriety software and maps, as well as secure data-sharing and free electronic tools and apps, are quick and cost-effective responses in times of crisis. In addition, reliable and accurate electronic databases of property and structures allow for rapid information retrieval, repair and rebuilding. Such systems can also help volunteers and first-responders in locating and assisting victims immediately after a catastrophic event, as well as facilitate longer-term recovery efforts by monitoring and coordinating activities to avoid waste, redundancy, bottlenecks and traffic congestion. ^2,3,4^

Natural disasters such as major earthquakes are tragic and unfortunate. However, out of crisis emerge opportunities for innovation and improvement. The ideas and proposals that result, are often bold and novel, and less constrained and shackled by policy, rules, regulations and norms. The Ezgeleh earthquake provides us with an opportunity to overcome shortcomings, learn more about crowdsourcing as a means of collecting information during a crisis, with the goal of reducing injury and mortality from future disasters in Iran. 
